# Bisphosphonate use in the horse: what is good and what is not?

**DOI:** 10.1186/s12917-019-1966-x

**Published:** 2019-06-24

**Authors:** Alexis Mitchell, Ashlee E. Watts, Frank H. Ebetino, Larry J. Suva

**Affiliations:** 1 Department of Veterinary Physiology and Pharmacology, College of Veterinary Medicine and Biomedical Sciences, College Station, TX USA; 2 Department of Large Animal Clinical Sciences, College of Veterinary Medicine and Biomedical Sciences, Texas A&M University, College Station, TX USA; 3 Department of Chemistry, University of Rochester, Rochester, NY USA

**Keywords:** Bisphosphonate, Bone resorption, Endocrinology-equine, Navicular syndrome

## Abstract

**Background:**

Bisphosphonates (BPs) are a family of molecules characterized by two key properties: their ability to bind strongly to bone mineral and their inhibitory effects on mature osteoclasts and thus bone resorption. Chemically two groups of BPs are recognized, non-nitrogen-containing and nitrogen-containing BPs. Non-nitrogen-containing BPs incorporate into the energy pathways of the osteoclast, resulting in disrupted cellular energy metabolism leading to cytotoxic effects and osteoclast apoptosis. Nitrogen-containing BPs primarily inhibit cholesterol biosynthesis resulting in the disruption of intracellular signaling, and other cellular processes in the osteoclast.

**Body:**

BPs also exert a wide range of physiologic activities beyond merely the inhibition of bone resorption. Indeed, the breadth of reported activities include inhibition of cancer cell metastases, proliferation and apoptosis in vitro. In addition, the inhibition of angiogenesis, matrix metalloproteinase activity, altered cytokine and growth factor expression, and reductions in pain have been reported. In humans, clinical BP use has transformed the treatment of both post-menopausal osteoporosis and metastatic breast and prostate cancer. However, BP use has also resulted in significant adverse events including acute-phase reactions, esophagitis, gastritis, and an association with very infrequent atypical femoral fractures (AFF) and osteonecrosis of the jaw (ONJ).

**Conclusion:**

Despite the well-characterized health benefits of BP use in humans, little is known regarding the effects of BPs in the horse. In the equine setting, only non-nitrogen-containing BPs are FDA-approved primarily for the treatment of navicular syndrome. The focus here is to discuss the current understanding of the strengths and weaknesses of BPs in equine veterinary medicine and highlight the future utility of these potentially highly beneficial drugs.

## Background

**Fig. 1:**
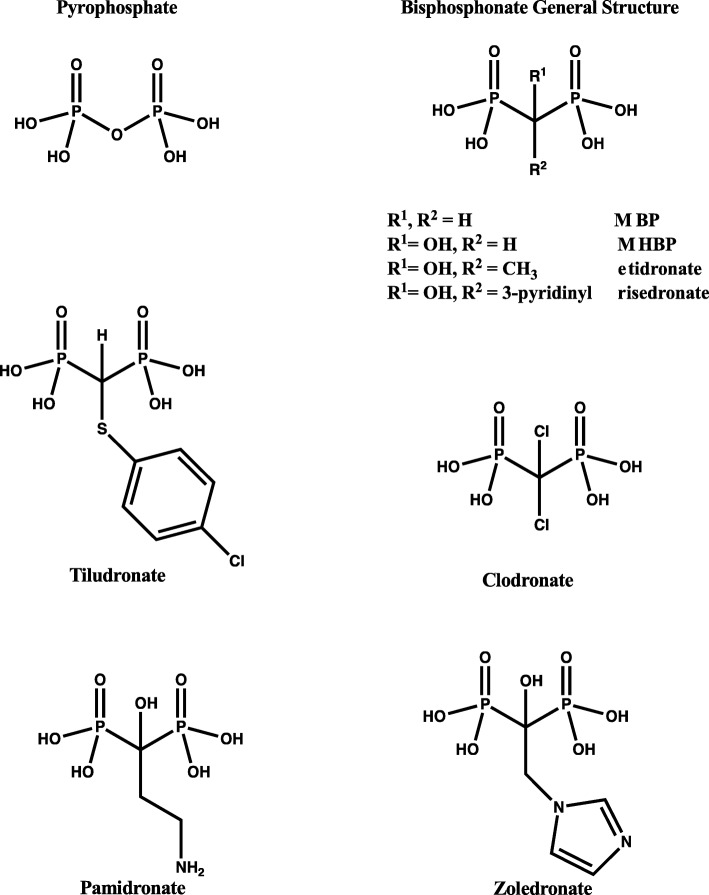
*Clinically-used bisphosphonates.* The general bisphosphonate chemical structure with potential subgroup substitutions is shown in comparison with endogenous pyrophosphate. Individual non-nitrogen bisphosphonate structures (Tiludronate and Clodronate) are shown in comparison to two of the nitrogen-containing bisphosphonate structures (Pamidronate and Zoledronate)

Bisphosphonates ((HO)_2_P(O)CR^1^R^2^P(O)(OH)_2_) (BPs) are chemically stable analogues of inorganic pyrophosphate (Fig. 1) that have been known to inhibit bone resorption since the 1960s [1, 2]. Indeed, it was studies on the role of inorganic pyrophosphate in the control of soft tissue and skeletal mineralization that resulted in the discovery of inhibitors of calcification that would resist hydrolysis by alkaline phosphatase [2]. The observation that inorganic pyrophosphate and BPs could not only inhibit the growth but also the dissolution of hydroxyapatite crystals drove further study of their ability to inhibit other physiologic processes, such as osteoclastic bone resorption [1–4].

BPs can be broadly classified into two groups (nitrogen and non-nitrogen containing), based on the presence or absence of an amine group and their distinct molecular modes of action [5]. The strong affinity of the BPs for the mineral phase of bone provides molecules with the unique property of selective uptake by bone to inherently provide a high degree of tissue specificity and facilitate BP access to osteoclasts. Furthermore, BPs tend to localize at the highest bone turnover sites due to greater exposed mineral at these surfaces where they can be taken up by osteoclasts during bone turnover. Within the osteoclast, the simpler, early generation, less potent non-nitrogen containing BPs (e.g.: tiludronate and clodronate) (Fig. 1) are metabolically incorporated into non-hydrolysable analogues of ATP, which interferes with ATP-dependent intracellular pathways [2, 6]. The more recently available and highly potent, nitrogen-containing BPs (such as pamidronate and zoledronate) (Fig. 1) are not metabolized as the non-nitrogen containing BPs but selectively inhibit farnesyl diphosphate synthase (FPPS) [7, 8], a key enzyme in the mevalonate/cholesterol biosynthetic pathway. In osteoclasts, disruption of tis pathway results in altered cellular processes such as ruffled border formation, critical for bone resorption [8, 9].

### What is the evidence for bisphosphonates efficacy in the horse?

BPs are Food and Drug Administration (FDA)-approved and commonly used in the US and Europe for the prevention and treatment of osteoporosis as well as to treat other bone diseases such as Paget’s disease and bone metastatic disease with remarkable efficacy in humans [10–13]. BPs significantly reduce the risk of hip or spine fractures in older women [10] and significantly improve the quality of life in patients with metastatic cancer to the bone [14]. Given the efficacy seen with the management of osteoporosis and metastatic bone disease, BP use has been explored in a myriad of other conditions. However, in the context of veterinary medicine, the primary use of BPs has been in the treatment of navicular syndrome in the horse [15, 16], as well as for palliative care of tumor bone pain in the dog [17]. Currently, two non-nitrogen containing BPs are FDA-approved and widely used in the treatment of navicular syndrome (tiludronate and clodronate; Fig. 1). Navicular syndrome is a chronic disease affecting the podotrochlear apparatus and is considered one of the most common causes of forelimb lameness in the horse [18]. In the US, both tiludronate and clodronate are approved for the control of clinical signs associated with navicular syndrome in horses. Any other veterinary use is considered off-label, and while not illegal, other uses have not been studied by either the manufacturers or the FDA. Both drugs are also labeled specifically for use in horses over the age of 4, an age at which bone remodeling naturally slows. To date, nitrogen containing BPs are not approved for use in the horse, but there are some reports of their use [19].

In the years since the widespread approved use of tiludronate disodium and clodronate in adult horses suffering from navicular syndrome, there have been reports of additional benefits of tiludronate use including the treatment of chronic back soreness [20] and lower hock osteoarthritis [21]. BPs are used in the horse in the treatment of chronic lameness due to many different causes, presumably, in part, due to the reported analgesic effects of BPs. Although blinded, these studies had clinical signs as the primary outcome measure and do not report any changes in bone mass. Interestingly bone mass has not been measured as an endpoint in any published equine study of BP safety or efficacy [22].

One of the oft stated goals of BP treatment in the horse is an increase in bone mass and strength, the result of a reduction in osteoclastic bone resorption, as observed in humans, but this parameter is largely unmeasured or ignored in equine studies [23]. Although a difficult endpoint in the equine setting, some consideration should be given to BMD measurement or perhaps more detailed evaluation of an appropriate bone mass surrogate, such as MRI, CT or serum bone turnover markers. Indeed, some of the positive outcomes reported following BP treatment may be due to the pain-relieving or anti-inflammatory effects of BP therapy and not the efficacy of BPs to inhibit bone resorption [24–26]. In this light, we recently reported the results of a small equine study in which the bone turnover markers C-terminal collagen-I telopeptide (CTX-I) and osteocalcin were measured following a single clodronate injection (IM) (1.4 mg/kg). Weekly blood draw and analysis revealed no significant effects on bone turnover markers, but did appear to reduce lameness [22]. These findings are consistent with the work of others [27] that showed tiludronate and clodronate (Fig. 1) do not appear to significantly impact bone tissue on a structural or cellular level using standard dose and administration schedules. In sum, these data support the notion that the effects of BP therapy in the horse may not be directly related to any inhibition of osteoclast activity.

In another interesting experimental paradigm, unilateral cast immobilization of the horse forelimb was used assess the protective effect of tiludronate on immobilization-induced bone loss [28]. Immobilization (disuse) increased levels of serum biomarkers of bone resorption that, as expected, were significantly reduced following tiludronate treatment at 1 mg/kg on days 0 and 28 of immobilization. Interestingly, this is one of the only studies directly demonstrating the anti-resorptive efficacy of tiludronate, or other BPs for that matter, in the horse. In general, equine-specific investigations of bone turnover and bone mass changes following BP treatment are lacking and sorely needed.

### That is important information, but what are the down sides?

Given the rampant BP use in the equine industry, there are only a few reports demonstrating a positive effect of either BP approved for use in horses with navicular syndrome [15, 16, 27] and none report bone-related complications. However there is a report that documented lack of change in bone resorption following tiludronate (1 mg/kg IV) or clodronate (1.8 mg/kg IM) treatment [27] as well as a lack of any significant change in serum markers of bone turnover following clodronate (1.4 mg/kg IM) treatment [22]. In contrast, the majority of human studies report both beneficial and not so beneficial effects of BP therapy in the treatment of postmenopausal osteoporosis and bone metastasis [9, 10, 12, 29–32]. The adverse events reported in humans, including an association with osteonecrosis of the jaw and perhaps the more troubling atypical fractures [33–38] may forewarn of concerns about BP use in the veterinary field. The lack of complications in veterinary BP literature could be due to the relatively low numbers of treated horses in these reports. Certainly, it was only after many years and many thousands of BP-treated human years that correlations between BP use and ONJ and AFFs were even recognized. It is important to note, it was only with the use of more potent nitrogen containing bisphosphonates that these adverse effects in small populations of patients have been observed and reported [39]. Despite these extremely rare complications, BPs remain a widely prescribed medication as BPs are proven to prevent fractures in patients with established osteoporosis or those who are at high risk of fracture. In these patients, the incidence of major complications associated with bisphosphonate use, such as ONJ and AFF, is very low [39]. It is important to place the potential negative effects of BP use alongside the advantages provided by BPs in the treatment of navicular syndrome and other disorders in veterinary medicine.

There has been much to do in the equine popular press highlighting recent human case reports and small clinical series where it has been suggested that long term bisphosphonate therapy (> 5 years) may suppress normal bone remodeling to such an extent that endogenous bone healing is decreased [40]. The ruckus is based on the concern that long term BP therapy would likely result in increased fracture risk and reduced fracture healing, if replicated in the equine setting. As discussed above, human BP-associated fractures result from suppressed bone turnover and are referred to as “atypical” because they occur at sites (e.g.: subtrochanteric femur) that are not typically associated with osteoporotic fractures [41]. With regard to fracture healing, because the remodeling phases of fracture healing involve significant elevations in bone resorption [42], and BPs significantly reduce bone resorption, there is interest in the possible utility of BPs to enhance fracture healing by preventing resorption of the mineralized fracture callus [43, 44]. Preclinical rodent [45], canine [46] and sheep [47] fracture repair studies provide evidence that BPs augment fracture healing resulting in stronger bone [45]. Interestingly, there are only two human clinical studies [44, 48] and none in the horse that have focused on this critical question.

In the HORIZON recurrent fracture clinical trial [48] no evidence of delayed fracture healing was observed when the BP (zoledronic acid; Fig. 1) treatment began within 90 days after hip fracture and no evidence of any delayed healing if treatment began within 2 weeks. More recently, the effects of early BP therapy on fracture healing and functional outcome following a fracture of the distal radius in osteoporotic patients was evaluated [49]. The fracture and bisphosphonates (FAB) trial was a double-blind, randomized, placebo-controlled trial involving 15 trauma centers across the United Kingdom that enrolled 421 bisphosphonate-naive patients aged ≥50 years with a radiographically confirmed fracture of the distal radius and randomized them in a 1:1 ratio to receive alendronate 70 mg once weekly (*n* = 215) or placebo (*n* = 206) within 14 days of the fracture. Administration of this highly potent N-containing BP did not affect fracture healing or clinical parameters [49]. Collectively, these data would contradict the anecdotal claims of many veterinary practitioners that the BPs mechanism of action disrupts the natural bone healing process. It is also possible that the potential for a catastrophic event is less likely in veterinary medicine as BP dosing is quite different. In the horse, non-N containing BPs tiludronate and clodronate (Fig. 1) are given in a single dose of 1 mg/kg IV and 1.8 mg/kg up to a maximum dose of 900 mg per horse, respectively every 3 months. In a recent human clinical trial, the same BP (clodronate) was given IM (200 mg/day for 10 days), approximately double the dose on a mg/kg basis and repeated 10-fold more for the treatment of active erosive osteoarthritis of the hand [50]. Indeed, the treating dose was even higher, since the patients also received a maintenance dose of clodronate IM (200 mg/day for 6 days after 3 and 6 months) [50]. This study demonstrated IM safety and efficacy with a significant reduction in the use of anti-inflammatory or analgesic drugs as well as increased hand functionality [50].

### In light of this expanding information, how should veterinarians use bisphosphonates in the future?

Given the growing concerns regarding treatment length and potential BP side effects, it is time for the veterinary community to push for more research and controlled trials of the use of the BPs, as well as focused and appropriate laboratory studies in the veterinary space. In addition, the incorporation of the existing human clinical data into the setting of CE as a means to advancing understanding of the utility and limitations of BP is warranted. Furthermore, studies with several second generation BPs may be required, given the distinct pharmacology and multiple subclasses of BPs that appear to act differently in mammalian assays and human clinical trials [51, 52].

Importantly, in view of the long half-life of BPs, it is feasible that BPs may have a significant effect on bone turnover after re-dosing, beyond the 3 monthly dose regimen currently approved in the horse. It is important to conduct additional well-designed dosing studies with appropriate bone end-points, such as imaging and serum markers of bone remodeling. Such studies are important as they may discriminate between the bone and non-bone effects of BPs and relieve concerns for adverse equine skeletal effects such as those that occur in human patients when there are significant and lasting reductions in CTX-I following BP treatment. In addition, veterinarians must consider the rationale for BP treatment. Since little evidence of changes in BMD or even bone strength changes exists following BPs in the horse, perhaps the primary utility of BP use is indeed the non-bone effect? This important distinction must be investigated.

The use of BP in the horse has been complicated of late with the recent public discourse regarding the off-label use of BPs in the yearling Thoroughbred industry. While the public outcry is concerned about ‘cleaning up’ potentially abnormal radiographs in young Thoroughbreds or change in fracture risk as the young Thoroughbred reach training and racing age, this is not supported by laboratory animal research. Early preclinical rodent studies of clodronate and etidronate (Fig. 1) convincingly and repeatedly demonstrated effects of non N-containing BPs (in doses from 0.1 to 10 mg/kg) in young growing rats with significant reductions in long bone length due to disruptions in endochondral ossification, but no differences in the mechanical properties of bone [53–55].

In humans, BPs are currently used in the treatment of pediatric bone disorders such as osteogenesis imperfecta (OI) [56], where any potential consequence at the growth plate is outweighed by the obvious patient benefits.. As a result of their efficacy, BPs are being increasingly used in other scenarios ranging in severity from spontaneous disuse fractures in patients with cerebral palsy [57] to the prevention of steroid-induced osteoporosis in ambulatory children [58] as well as the prevention of bone loss in children with hypercalciuria [59]. In these cases, the beneficial effects of BPs outweigh the potential negative effects on endochondral ossification and long bone growth [60]. Importantly, the doses used are significantly greater than the doses currently approved for use in the adult horse.

Certainly, in the setting of OI, cyclical BPs transiently reduce pain and improve function [61]. Doses of the N-containing BP (zoledronic acid) were 1.1 mg/kg every 3 months (ages 2–3) and patients > 3 years of age, 1.5 mg/kg/dose every 4 months (maximum dose ≤45 mg/infusion and 4.5 mg/kg/year) [61]. In these patients, pain relief occurred immediately following infusion with functional improvements observed 4 weeks later [61]. However, both pain and physical function return to pretreatment levels by the subsequent infusion, suggesting a potential non-osteoclast-mediated mechanism for improved pain relief.

With regard to the apparent analgesic effects of BPs, at least in humans, the data would suggest these are more likely to be associated with N-containing BPs although little or no mechanistic understanding exists. There is a study examining the BP analgesic effect from a meta-analysis of 8595 patients enrolled in a number of BP clinical trials [62]. Twenty-two (79%) of the 28 placebo-controlled trials found no analgesic benefit for BPs. The authors conclude that N-containing BPs appear to be beneficial in preventing pain by delaying the onset of bone pain (in the oncology setting) rather than by eliciting an analgesic effect per se [62]. In contrast, others have suggested that N-containing BPs are metabolized to novel ATP analogs facilitating activation of ATP-gated P2X receptors, albeit in rat sensory neurons, as a potential analgesia mechanism [63]. On the other hand, Kim *et. al*. [64] compared the analgesic activity of a variety of N-containing and non N-containing BPs in mice. The results suggest that non N-containing BPs, not N-containing BPs, display analgesic effects at doses lower than those inhibiting bone resorption, similar to what we have reported in the horse [22]. Although the jury is still out regarding the specific mechanism(s) responsible for BP-induced analgesia, the best in vivo evidence for BP-associated analgesic effect may well be with non N-containing BP in the horse [22].

## Conclusions

In the horse there is currently a dearth of information regarding the effect of single and repeated doses of clodronate and tiludronate. Well-designed and appropriately powered research by non-biased researchers with germane bone parameters as outcome measures must be completed. Only with this data can horse owners and practitioners alike make informed decisions regarding the efficacy and appropriate clinical use of these potent molecules. Certainly, clients and practitioners alike require ongoing educational efforts regarding the efficacy and appropriate clinical use of these potent molecules. Following the development of a better understanding of BP effects in the horse, appropriately designed and powered placebo-controlled studies will determine to what extent beneficial BP effects on lameness are due to the inhibition of bone resorption and ascertain the details of repeat dosing in the equine setting. Such a strategy is required to ensure safer clinical use and produce a sufficient level of evidence to ensure safety.

## Data Availability

Not applicable, no primary data presented.

## Abbreviations

AFF: Atypical femoral fractures
BP: Bisphosphonates
CTX-I: C-terminal collagen-I telopeptide
FDA: Food and Drug Administration
FPPS: Farnesyl diphosphate synthase
OI: Osteogenesis imperfecta
ONJ: Osteonecrosis of the jaw
